# Supplementary material for: Influence of Spirituality and Religiosity of Cancer Patients on Their Quality of Life
Source: Int J Environ Res Public Health. 2022 Apr 19;19(9):4952. doi: 10.3390/ijerph19094952 (PMC9103221; doi:10.3390/ijerph19094952)
Supplement: Supplementary file 1 [file ijerph-19-04952-s001.zip › ijerph-1661429-supplementary.pdf]

1. Daily Spiritual Experience Scale – DSES

The list that follows includes items you may or may not experience. Please consider how often you directly have this experience, and try to disregard whether you feel you should or should not have these experiences. A number of items use the word 'God' If this word is not a comfortable one for you, please substitute another word that calls to mind the divine or holy for you.

Questions	Never	Once in a while	Some days	Most days	Every day	Many times a day
1. Have you been spiritually touched by the beauty of creation?						
2. Have you felt God's presence, or the presence of the divine or holy?						
3. Have you experienced a connection to all of life?						
4. Have you felt close to God, or to the divine or transcendent as expressed in other words?						
5. Have you desired to be closer to God or in union with the divine?						
6. Have you felt God's love or divine love for you directly?						
7. Have you felt God's love or compassionate love for you through others?						
8. Have you felt a selfless caring for others?						
9. Have you accepted others even when they have done things you think are wrong?						
10. Have you found strength in your spirituality or religion?						
11. Have you found comfort in your spirituality or religion?						
12. Have you felt guided by God in the midst of daily activities?						
13. Have you asked for God's help in the midst of daily activities.						
14. During worship, or at other times when connecting with God, have you felt joy that lifts you out of your daily concerns?						
15. Have you felt thankful for your blessings?						

Question	Not at all	Somewhat close	Very close	As close as possible
16. Have you felt deep inner peace or harmony?				

2. EORTC QLQ-C30 questionnaire (version 3)

We are interested in some things about you and your health. Please answer all of the questions yourself by circling the number that best applies to you. There are no "right" or "wrong" answers. The information that you provide will remain strictly confidential.

Questions	Not at all	A little	Quite a bit	Very much
1. Do you have any trouble doing strenuous activities, like carrying a heavy shopping bag or a suitcase?				
2. Do you have any trouble taking a long walk?				
3. Do you have any trouble taking a short walk outside of the house?				
4. Do you need to stay in bed or a chair during the day?				
5. Do you need help with eating, dressing, washing yourself or using the toilet?				

During the past week:

Questions	Not at all	A little	Quite a bit	Very much
6. Were you limited in doing either your work or other daily activities?				
7. Were you limited in pursuing your hobbies or other leisure time activities?				
8. Were you short of breath?				
9. Have you had pain?				
10. Did you need to rest?				
11. Have you had trouble sleeping?				
12. Have you felt weak?				
13. Have you lacked appetite?				
14. Have you felt nauseated?				
15. Have you vomited?				
16. Have you been constipated?				
17. Have you had diarrhea?				
18. Were you tired?				
19. Did pain interfere with your daily activities?				
20. Have you had difficulty in concentrating on things, like reading a newspaper or watching television?				
21. Did you feel tense?				

Questions	Not at all	A little	Quite a bit	Very much
22. Did you worry?				
23. Did you feel irritable?				
24. Did you feel depressed?				
25. Have you had difficulty remembering things?				
26. Has your physical condition or medical treatment interfered with your family life?				
27. Has your physical condition or medical treatment interfered with your social activities?				
28. Has your physical condition or medical treatment caused you financial difficulties?				

For the following questions please circle the number between 1 and 7 that best applies to you

29. How would you rate your overall health during the past week?

1	2	3	4	5	6	7
Very poor						Excellent

30. How would you rate your overall quality of life during the past week?

1	2	3	4	5	6	7
Very poor						Excellent

3. EORTC QLQ-FA12 questionnaire

Patients sometimes report that they have the following symptoms or problems. Please indicate the extent to which you have experienced these symptoms or problems during the past week. Please answer by circling the number that best applies to you.

During the past week:

Questions	Not at all	A little	Quite a bit	Very much
1. Have you lacked energy?				
2. Have you felt exhausted?				
3. Have you felt slowed down?				
4. Did you feel sleepy during the day?				
5. Did you have trouble getting things started?				
6. Did you feel discouraged?				
7. Did you feel helpless?				
8. Did you feel frustrated?				
9. Did you have trouble thinking clearly?				
10. Did you feel confused?				
11. Did tiredness interfere with your daily activities?				
12. Did you feel that your tiredness is (was) not understood by the people who are close to you ?				

Self-constructed questionnaire

1. Sex
   a) Woman
   b) Man

2. Please provide your age ……………………………………………

3. Where do you life?
   a) Countryside
   b) City below 20.000 resident
   c) City from 20.000 up to 100.000 residents
   d) A city with over 100.000 residents

4. In which voivodship do you live?
   a) Lesser Poland
   b) Podkarpackie Province
   c) Other (what?) ……………………………………………………………………..

5. What is your marital status?
   a) Miss / Bachelor
   b) Married / Married
   c) Widow / Widower
   d) Divorced / Divorced
   e) Separated / Separated

6. What is your education?
   a) Basic
   b) High school
   c) Basic vacational
   d) Avarage
   e) High

7. What type of cancer have you been diagnosed with?
……………………………………………………………………………………………….

8. What kind of treatment have you been receiving? (you can choose several answers)
   a) Operational
   b) Chemotherapy
   c) Radiotherapy
   d) Immunotherapy
   e) Hormone therapy
   f) With the use of nuclear medicine
   g) Other (what?) …………………………………………………………………………

9. Are you currently undergoing oncological treatment?
   a) Yes
   b) No (If you marked this answer, please answer question 10)

10. How long did the treatment take? …………………………………

11. Are you a believer: yes no

If so, what religion are you ....
